# Distribution of dementia severity and its functional correlates in community-dwelling older adults in Vietnam

**DOI:** 10.1186/s12877-026-07588-3

**Published:** 2026-05-02

**Authors:** Huong Thu Vu, Anh Trung Nguyen, Hung Trong Nguyen

**Affiliations:** 1 Hanoi Medical University, Hanoi, Vietnam; 2 National Geriatric Hospital, Hanoi, Vietnam

**Keywords:** Dementia, Clinical Dementia Rating, Instrumental Activities of Daily Living, Quality of life, Older adults, Vietnam

## Abstract

**Background:**

Dementia is a growing public health concern in low- and middle-income countries (LMICs), where population aging is accelerating and evidence on disease severity remains limited. Vietnam is undergoing a rapid demographic transition, yet community-based evidence on dementia severity and its associated factors remains limited.

**Methods:**

We conducted a cross-sectional study among 399 community-dwelling older adults (≥ 60 years) diagnosed with dementia in Hai Duong province, Vietnam. Dementia severity was assessed using the Clinical Dementia Rating (CDR) scale. Sociodemographic characteristics, physical activity, social engagement, depressive symptoms, neuropsychiatric symptoms, instrumental activities of daily living (IADL), and quality of life (EQ-5D-5L) were collected. Multivariable Poisson regression with robust variance was used to identify factors associated with dementia severity.

**Results:**

Overall, 43.3% of participants had very mild and mild dementia, 30.1% had moderate dementia, and 26.6% had severe dementia. In univariate analyses, several sociodemographic, clinical, and behavioral factors were associated with severe dementia. However, after adjustment in the multivariable model, higher IADL scores (PR = 0.69; 95% CI: 0.56–0.84) and higher EQ-5D-5 L scores (PR = 0.92; 95% CI: 0.87–0.97 per 0.1 increase) were associated with a lower prevalence of severe dementia.

**Conclusion:**

This study provides community-based evidence on dementia severity among older adults in two districts of Hai Duong province, Vietnam, showing that more than half of participants had moderate-to-severe dementia. Functional independence and quality of life were associated with dementia severity and may represent important considerations when designing community-based dementia care strategies.

**Supplementary Information:**

The online version contains supplementary material available at 10.1186/s12877-026-07588-3.

## Introduction

Dementia is a chronic progressive syndrome characterized by a decline in multiple cognitive domains, leading to loss of autonomy in daily functioning [1]. It is currently the seventh leading cause of death worldwide and one of the major causes of disability and dependency among older adults [1]. Globally, the number of people living with dementia is projected to increase from 57.4 million in 2019 to 152.8 million by 2050 [2]. This condition affects not only patients but also caregivers, families, and communities, imposing a substantial financial and social burden on healthcare systems. The global cost of dementia care was estimated at USD 800 billion (1% of global GDP) in 2015, and is expected to reach USD 2 trillion by 2030 [3].

Vietnam is no exception to this global health trend. According to the 2019 Population and Housing Census, the country had 11.41 million older adults, accounting for 11.86% of the total population, and this proportion is projected to reach 27.11% (31.69 million people) by 2069 [4]. Such demographic transition has led to a shift in disease patterns, with a growing burden of non-communicable diseases, including dementia.

A recent systematic review that screened over 14,000 articles and included 24 eligible studies from 122 low- and middle-income countries (LMICs) estimated the annual per-person cost of dementia care to range from USD 590.78 for mild dementia to USD 25,510.66 for severe dementia [5]. This highlights how increasing dementia severity substantially drives healthcare and caregiving costs. The Clinical Dementia Rating (CDR) scale is widely used to classify dementia severity into very mild, mild, moderate, and severe stages [6]. Several factors, such as age, sex, educational attainment, depression, functional independence in daily living, comorbidities, and quality of life have been found to be associated with dementia severity [7–11].

In Vietnam, however, community-based evidence describing the distribution of dementia severity and its associated factors remains limited. Therefore, this study aimed to describe the distribution of dementia severity and to identify factors associated with severe dementia among community-dwelling older adults in Vietnam. The findings may provide community-based evidence to inform future research and community-based care strategies aimed at supporting daily functioning and quality of life among older adults living with dementia.

## Materials and methods

### Study population

- Inclusion criteria were:

- Individuals aged 60 years or older;
- Residing in Thanh Mien and Gia Loc districts, Hai Duong province, Vietnam;
- Diagnosed with dementia by neurologists/geriatricians at the National Geriatric Hospital and Hai Duong Provincial General Hospital using DSM-5 diagnostic criteria [6, 12];
- Provided voluntary informed consent to participate.

- Exclusion criteria were:

- Presence of severe acute illness or impaired consciousness at the time of assessment;
- Declined to participate or withdrew from the study.

### Study setting and duration

The study was conducted from July 2021 to December 2022 in Thanh Mien and Gia Loc districts, Hai Duong province. Hai Duong is located in the Red River Delta region and includes both rural and peri-urban communities. The province reflects common socio-demographic characteristics of northern Vietnam but may not fully represent the national population of Vietnam. The study sites were selected based on the long-standing clinical and research collaboration between local health facilities and the National Geriatric Hospital [13, 14].

### Sample size

The sample size was calculated using the WHO-recommended formula for estimating a population proportion [15]:

$$\mathrm{n}={\mathrm{Z}}_{1-{\upalpha\:}/2}^{2}\:\times\:\:\frac{\mathrm{P}(1-\mathrm{P})}{{\left({\upepsilon\:}\mathrm{P}\right)}^{2}}$$

Where:

- P: estimated prevalence of severe dementia among older adults, set at 39.3%, based on Moon et al. (2016) [11];
- $\:{Z}_{1-{\upalpha\:}/2}$: 1.96, corresponding to a 95% confidence level;
- $$\:\epsilon\:$$: relative precision: 0.13.

The minimum required sample size was estimated to be 375 participants after accounting for 10% potential non-response. A total of 399 participants were included in the study.

### Study design

This was a cross-sectional study utilizing baseline data from the REACH-VA project (“Enhancing dementia caregiver support interventions and research capacity in Vietnam”) implemented in the two study districts.

### Sampling procedure

1. Administrative approval was obtained from district health authorities.
2. All commune health stations within each district were listed, and communes were randomly selected.
3. Lists of older adults residing in selected communes were obtained from local administrative records.
4. Local health staff screened older adults for potential cognitive impairment (memory, behavioral, or language changes) during routine community health activities. A total of 499 individuals suspected of cognitive impairment were identified and invited for clinical assessment.
5. Individuals identified were invited for clinical assessment conducted by trained geriatric specialists. Dementia was diagnosed according to DSM-5 criteria, combined with neurological examination, Mini-Cog testing, and Clinical Dementia Rating (CDR) scoring.

**Fig. 1:**
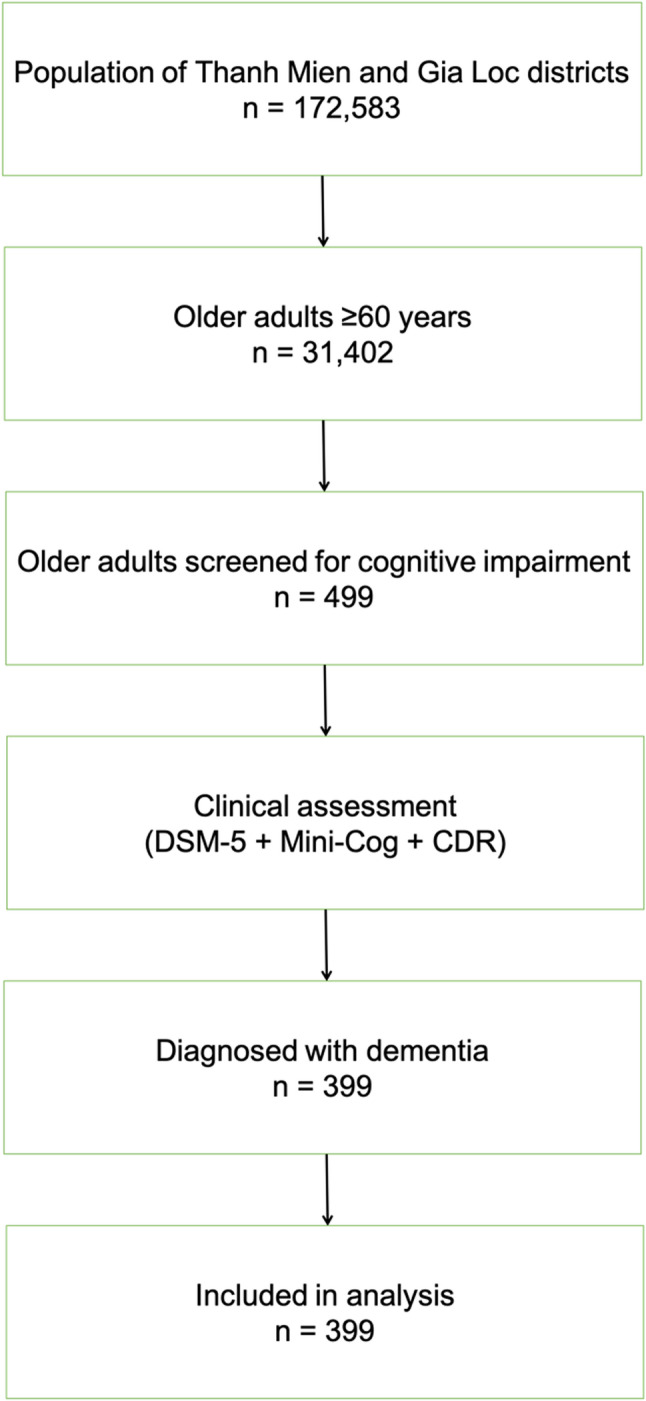
Flowchart of participant selection for the community-based dementia study conducted in Thanh Mien and Gia Loc districts, Hai Duong province

The sampling and participant selection process is shown in Fig. 1.

### Measures

- Dementia Severity: Dementia severity was assessed using the Vietnamese version of the Clinical Dementia Rating (CDR) scale [6]. This instrument is widely used globally to categorize dementia severity. The CDR consists of two parts: Part 1 is completed by an informant (family member or caregiver) and Part 2 is administered to the patient. Information from both parts is compared to evaluate six domains: memory, orientation, judgment and problem-solving, community affairs, home and hobbies, and personal care. Both component scores and the global CDR score were used for classification. The CDR is scored in five levels: 0 (no dementia), 0.5 (very mild dementia), 1 (mild dementia), 2 (moderate dementia), and 3 (severe dementia). For the primary analysis, dementia severity was dichotomized as severe dementia (CDR = 3) versus non-severe dementia (CDR < 3) to facilitate clinical interpretation by distinguishing individuals with advanced cognitive and functional impairment from those in earlier stages.
- Sociodemographic Characteristics: These included age, sex, educational level, marital status, and employment status.
- Physical Activity: Physical activity levels were assessed using the International Physical Activity Questionnaire – Short Form (IPAQ-SF). The IPAQ-SF evaluates the type, frequency, intensity, and duration of physical activity. The Vietnamese version has been validated [16]. Metabolic equivalent (MET)-minutes per week were calculated by multiplying minutes spent in each activity by MET values (8 for vigorous, 4 for moderate, and 3.3 for walking), and then summed across activity domains.
- Social Engagement: Frequency of participation in social activities and frequency of visiting friends or neighbors were self-reported as the number of times per week or month.
- Instrumental Activities of Daily Living (IADL): Higher-level functioning was evaluated using the Instrumental Activities of Daily Living (IADL) scale. The IADL includes eight items: telephone use, shopping, food preparation, housekeeping, laundry, transportation, medication management, and financial management. The Vietnamese version has been used [17]. Scores range from 0 (dependent) to 8 (independent), with a cut-off score of 4 to classify independence.
- Depressive Symptoms: Depressive symptoms were assessed using the 15-item Geriatric Depression Scale (GDS-15). The Vietnamese version has been validated [18]. Total scores range from 0 to 15; scores 0–5 indicate normal mood, while scores 6–15 suggest depressive symptoms.
- Neuropsychiatric Symptoms: Neuropsychiatric symptoms were assessed using the Neuropsychiatric Inventory (NPI). The NPI evaluates both symptom severity and caregiver distress across 12 domains, including delusions, hallucinations, agitation/aggression, depression/dysphoria, anxiety, euphoria, apathy, disinhibition, irritability, aberrant motor behavior, sleep disturbances, and appetite changes. The Vietnamese version (V-NPI) has been validated and shown to be reliable in previous studies [19]. Higher scores indicate greater symptom severity and caregiver burden.
- Quality of Life: Quality of life was assessed using the Vietnamese version of the EQ-5D-5L [20], which measures five dimensions: mobility, self-care, usual activities, pain/discomfort, and anxiety/depression. Each dimension has five response levels from no problem [1] to extreme difficulty [5]. Combined ratings form a health state that is converted into a utility index score ranging from 0 (worst) to 1 (best).

### Data management and statistical analysis

Data were entered using KoboToolbox and analyzed with Stata 17.0.

Categorical variables were summarized as frequencies and percentages; continuous variables as means (± SD) or medians (IQR), depending on distribution.

- Group comparisons used Chi-square/Fisher’s exact tests for categorical variables and t-test/ANOVA or Kruskal–Wallis tests for continuous variables.
- Univariable Poisson regression analyses were first performed to examine associations between potential factors and severe dementia. Variable selection followed a sequential, pre-specified strategy. Age and sex were entered as a priori confounders in all models irrespective of statistical significance. All remaining candidate variables — identified from prior literature and available in the baseline dataset — were first examined in minimally adjusted models (Supplementary Table S2). Some additional variables collected as part of the broader REACH-VN baseline assessment (e.g., sleep quality, alcohol use, and smoking status) were explored in preliminary analyses (Supplementary Table S2) but were not included in the final multivariable model because they were not part of the primary conceptual framework of this study. This approach avoided data-driven variable selection and ensured that the final model reflected theoretical rather than empirical prioritisation. No missing data were observed for any variables included in the analysis; therefore, all participants were included in the complete-case analysis. Multicollinearity was assessed via variance inflation factors (mean VIF 1.47; all values < 2.10), and results are presented in Supplementary Table S3.
- For the primary analysis, dementia severity was dichotomised as severe (CDR = 3) versus non-severe (CDR < 3); given the high prevalence of both moderate (30.1%) and severe (26.6%) dementia and the demonstrated violation of the proportional odds assumption in ordinal logistic regression (Brant test *p* < 0.001), Poisson regression with robust variance was used to directly estimate prevalence ratios, avoiding the overestimation inherent to odds ratio-based models in cross-sectional studies with common outcomes; multinomial and ordinal logistic regression results are presented as sensitivity analyses in Supplementary Tables S4 and S5 [21, 22]. These additional analyses were conducted to assess the robustness of the findings when alternative modeling approaches for the ordinal CDR outcome were considered.
- A *p*-value < 0.05 was considered statistically significant.

### Ethical considerations

The study complied with the Declaration of Helsinki and was approved by the Hanoi Medical University Ethics Committee (Approval No. 476/GCN-HĐĐĐNCYSH-ĐHYHN, dated 23/07/2021).

All participants provided written informed consent to participate in the study.

Clinical trial number: not applicable.

## Results

**Table 1:** General characteristics of participants (*N* = 399)

Characteristics	*n* (%) / Mean ± SD
Age (years)	79.6 ± 10.3
Female	289 (72.4)
Educational level	
No formal education	232 (58.1)
Primary school	69 (17.3)
Secondary school or higher	98 (24.6)
Occupation before retirement	
Governmental staff	55 (13.8)
Farmer	293 (73.4)
Other (worker, freelance, etc.)	51 (12.8)
Marital status	
Married and living with spouse	244 (61.2)
Widowed / single / divorced	155 (38.8)
BMI category	
Underweight	98 (24.6)
Normal	278 (69.7)
Overweight	23 (5.8)

As shown in Table 1, among 399 participants, 289 (72.4%) were female, with a mean age of 79.6 ± 10.3 years. Most participants had no formal education or only primary schooling (75.4%), were farmers before retirement (73.4%), and had a normal BMI (69.7%). Nearly 40% were widowed, single, or divorced.

**Fig. 2:**
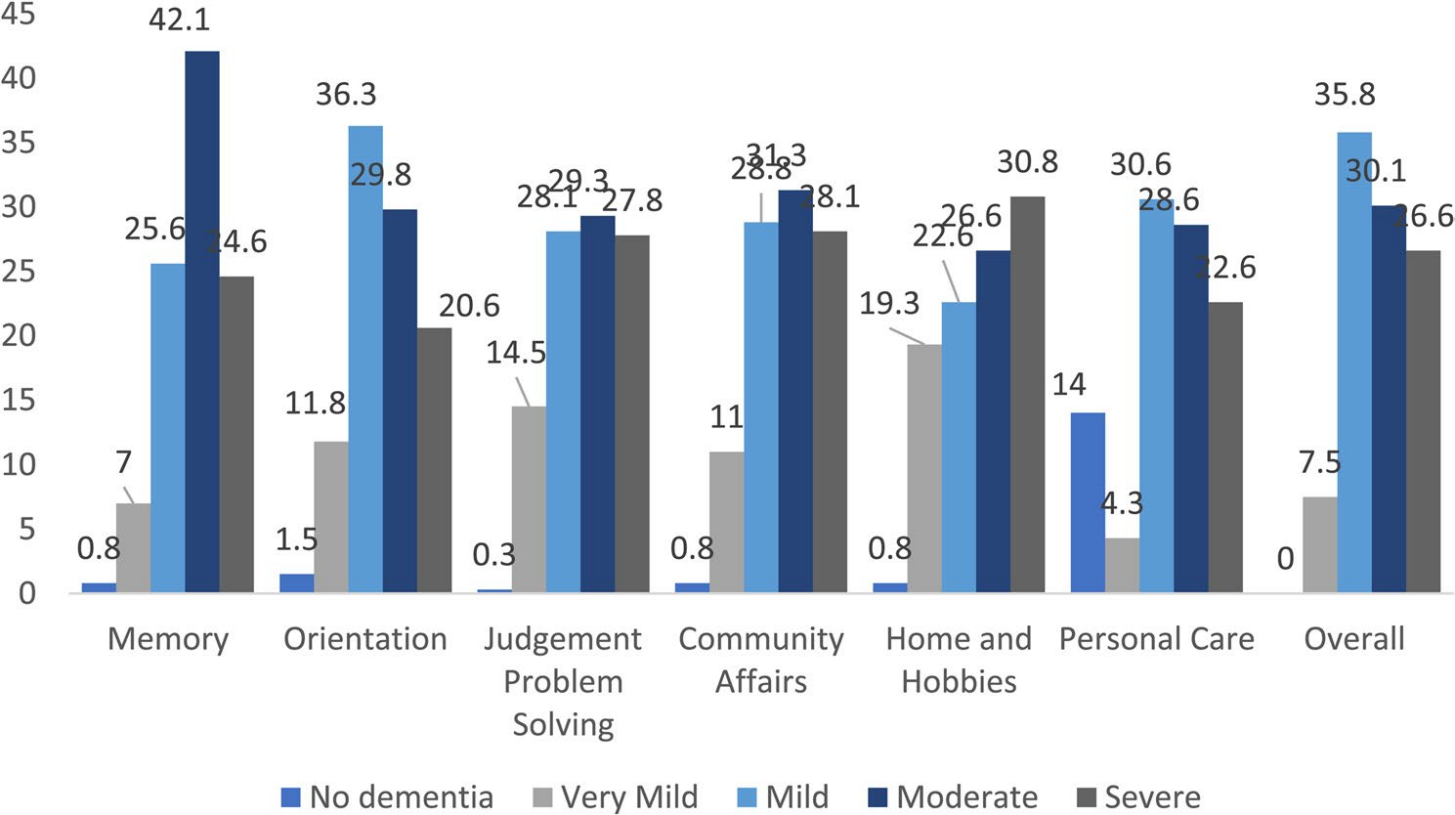
Distribution of dementia severity according to the CDR scale

As shown in Figs. 2, 43.3% of participants had very mild or mild dementia, 30.1% had moderate dementia, and 26.6% had severe dementia. Thus, more than half of participants had moderate or severe dementia (56.7%).

**Table 2:** Factors associated with severe dementia (univariate analysis)

Factors	PR (95% CI)	*p*-value
Age (per 1-year increase)	1.06 (1.04–1.09)	**< 0.001**
No formal education (vs. ≥secondary school)	3.13 (1.67–5.88)	**< 0.001**
Farmer (vs. governmental staff)	2.78 (1.22–6.36)	**0.01**
Widowed/single/divorced (vs. married)	2.70 (1.82–4.01)	**< 0.001**
Underweight (vs. normal BMI)	1.69 (1.12–2.51)	**0.03**
Depressive symptoms	2.57 (1.64–4.03)	**< 0.001**
Behavioral and psychological symptoms	3.32 (1.61–6.82)	**< 0.001**
High physical activity (vs. low)	0.19 (0.07–0.52)	**< 0.001**
Frequency of social activities (per month)	0.59 (0.46–0.76)	**< 0.001**
Frequency of visiting friends/neighbors (per month)	0.78 (0.69–0.88)	**< 0.001**
Quality of life (EQ-5D-5L per 0.1 score increase)	0.84 (0.81–0.88)	**< 0.001**
Instrumental Activities of Daily Living (per score increase)	0.60 (0.50–0.71)	**< 0.001**

High physical activity defined as ≥ 600 MET-minutes/week according to the IPAQ-SF classification. Depressive symptoms defined as GDS-15 score ≥ 6. Behavioral and psychological symptoms assessed using the Neuropsychiatric Inventory (NPI)

IADL scores range from 0 to 8, with higher scores indicating greater independence. EQ-5D-5L utility score ranges from 0 to 1, with higher scores indicating better quality of life

Abbreviations: *PR* Prevalence ratio, *CI *Confidence interval

Bold values indicate statistical significance (*p* < 0.05)

As shown in Table 2, univariate analysis revealed that several factors were significantly associated with severe dementia. Older age, lower education, farming occupation, being widowed or single, underweight status, depressive symptoms, and behavioral or psychological symptoms were associated with severe dementia in the univariate analyses. In contrast, higher physical activity, greater social engagement, better quality of life, and higher functional independence were associated with lower prevalence of severe dementia in univariate analyses (all *p* < 0.05).

**Table 3:** Adjusted associations with severe dementia from the multivariable Poisson regression with robust variance model

Factors	Adjusted PR (95% CI)	*p*-value
Female (vs. male)	0.97 (0.62–1.52)	0.90
Age (per 1-year increase)	1.02 (0.99–1.05)	0.18
Widowed/single/divorced (vs. married)	1.39 (0.87–2.24)	0.17
Frequency of visiting friends/neighbors (per month)	0.94 (0.85–1.03)	0.16
Physical activity (MET-min/week)	1.00 (0.99–1.00)	0.12
Depressive symptoms (GDS-15 score)	1.04 (0.98–1.11)	0.19
Instrumental activities of daily living (per score increase)	0.69 (0.56–0.84)	< 0.001
Quality of life (EQ-5D-5L per 0.1 score increase)	0.92 (0.87–0.97)	0.004

As shown in Table 3, in the multivariable Poisson regression model, only instrumental activities of daily living and quality of life remained significantly associated with severe dementia. Each one-point increase in the IADL score was associated with a lower prevalence of severe dementia (adjusted PR = 0.69; 95% CI: 0.56–0.84). Similarly, higher quality of life scores was associated with a lower prevalence of severe dementia (adjusted PR = 0.92; 95% CI: 0.87–0.97).

## Discussion

This study provides community-based evidence on the distribution of dementia severity among older adults in two districts of Hai Duong province, Vietnam. The findings have important implications for healthcare policy, particularly in prioritizing health services for the aging population. More than half of older adults living in the community presented with moderate to severe dementia, and approximately one-fourth were classified as severe, reflecting the substantial disease burden in Vietnam. Our findings are consistent with those reported by Herrera et al. in a community-based cohort in Brazil, where severe dementia accounted for 24% of cases [23]. However, the proportion of severe dementia in our study was lower than that reported by Marra et al. (2007), who found a prevalence of 45.56% [24] and the 18.5% and 10.9% reported in South Korea for CDR scores of 2 and 3, respectively [10]. These discrepancies may be explained by differences in healthcare systems, dementia awareness, and screening programs. While the United States and South Korea have established early detection programs, Vietnam remains a lower-middle-income country where awareness and early detection of dementia remain limited.

In the univariate analyses, several sociodemographic, clinical, and behavioral factors—including age, educational attainment, marital status, depressive symptoms, physical activity, and social engagement were associated with severe dementia. However, after simultaneous adjustment in the multivariable model, only functional independence (IADL) and quality of life remained independently associated with dementia severity. This pattern suggests that many associations observed in univariate analyses may reflect indirect relationships or shared underlying pathways rather than independent effects. Sociodemographic and psychosocial factors may influence dementia severity through their relationships with functional status, overall health, and well-being.

Several demographic and health-related factors, including age, educational attainment, marital status, and nutritional status, were associated with severe dementia in univariate analyses. Similar patterns have been reported in previous studies, where older age, lower education, social vulnerability, and poor nutritional status were linked with greater dementia severity [11, 25–27]. In the present study, these associations were attenuated after multivariable adjustment, suggesting that their relationships with dementia severity may operate indirectly through functional decline and overall health status.

Depressive symptoms were also associated with severe dementia in the univariate analysis. Depression is common among individuals with dementia and may reflect both neuropsychiatric manifestations of the disease and psychosocial consequences of cognitive decline [28, 29]. In the present study, the association was attenuated after multivariable adjustment, suggesting that depressive symptoms may be linked to dementia severity through related factors such as reduced functional independence and poorer quality of life. Sensitivity analyses using multinomial and ordinal logistic regression produced broadly consistent patterns, with functional independence and quality of life remaining strongly associated with greater dementia severity.

Lower levels of physical activity and reduced social engagement were also associated with severe dementia in the univariate analyses. Previous studies have reported similar associations between physical activity, social participation, and cognitive health in older adults [30, 31]. Potential mechanisms proposed in the literature include improved cerebral blood flow, enhanced neuroplasticity, and cognitive stimulation related to physical and social engagement [32, 33]. In the present study, these associations were attenuated after multivariable adjustment, suggesting that they may reflect indirect relationships with dementia severity.

The multivariable analysis in this study identified two factors that remained independently associated with severe dementia: functional independence in instrumental activities of daily living (IADL) and quality of life. Specifically, each one-point increase in the IADL score was associated with a lower prevalence of severe dementia. This finding is consistent with the results of Marra et al., who demonstrated a significant association between dementia severity and functional independence using the Brazilian ADL and IADL scales (*p* < 0.001). The correlation between IADL scores and dementia severity was strong across the full sample (*p* < 0.0001; *r* = − 0.818), as well as within subgroups of mild dementia (*p* = 0.007; *r* = − 0.530) and severe dementia (*p* < 0.0001; *r* = − 0.723) [24]. Furthermore, IADL was found to decline earlier in the course of dementia, while basic ADL impairment was more pronounced in later stages [24]. This aligns with prior evidence indicating that functional disability is closely linked to dementia progression [17, 34]. Individuals in advanced stages experience increasing difficulty performing even basic daily activities, resulting in loss of independence and greater reliance on caregivers [35]. The extent of loss of functional autonomy therefore reflects a patient’s diminishing adaptive capacity in everyday life and serves as an important clinical indicator of disease severity. Functional independence therefore appears to be closely associated with dementia severity and may reflect the functional consequences of cognitive decline [36]. These findings highlight the close association between functional independence, quality of life, and dementia severity.

In the multivariable model, each 0.1 increase in the EQ-5D-5L utility score was associated with an 8% lower prevalence of severe dementia (PR = 0.92). Given that the EQ-5D-5L score ranges from 0 to 1, expressing the association per 0.1 increase provides a clinically interpretable measure of change in quality of life. This finding is consistent with the study by Hanna Maria Roitto et al. (2019), which demonstrated a significant association between quality of life and dementia severity among 352 individuals with dementia [37]. Patients with severe dementia (CDR = 3) exhibited poorer mobility, visual function, eating ability, language function, daily self-care, and greater behavioral disturbances compared with those with mild to moderate dementia (CDR < 3) [37]. As a result, individuals with advanced dementia tend to experience significantly reduced quality of life.

This study has several limitations. First, the cross-sectional design precludes establishing causal relationships between the identified factors and dementia severity. Reduced functional independence and poorer quality of life may contribute to greater dementia severity, but they may also represent consequences of more advanced disease. Second, the study was conducted in two districts of Hai Duong province, which may limit the generalizability of the findings to the broader Vietnamese population. Nevertheless, the study provides valuable community-based evidence from a Vietnamese setting where epidemiological data on dementia severity remain limited. Third, potential measurement overlap may exist between the outcome and one of the predictors. The Clinical Dementia Rating (CDR) scale includes domains such as community affairs and home activities, which are conceptually related to instrumental activities of daily living (IADL). Therefore, the strong association observed between IADL and dementia severity may partly reflect overlapping measurement constructs rather than a purely independent relationship.

## Conclusion

In this community-based sample of older adults with dementia in Hai Duong province, more than half of participants (56.7%) had moderate to severe disease. Several sociodemographic, clinical, and behavioral factors were associated with severe dementia in univariate analyses. However, after adjustment, only lower functional independence in instrumental activities of daily living and poorer quality of life remained independently associated with severe dementia. These findings highlight the close relationship between functional independence, quality of life, and dementia severity and may provide useful considerations for community-based dementia care programs.

### Recommendations

Targeted screening and community-based interventions may help support older adults living with dementia. Future longitudinal studies are needed to clarify the causal relationships between functional decline, quality of life, and dementia severity. Additionally, training primary healthcare workers and caregivers in early recognition and effective care strategies is essential to reduce the growing burden of dementia in the context of Vietnam’s rapidly aging population.

## Supplementary Information

Supplementary Material 1.

## Data Availability

The datasets used and/or analyzed during the current study are available from the corresponding author on reasonable request.

## Abbreviations

ADL: Activities of Daily Living
IADL: Instrumental Activities of Daily Living
CDR: Clinical Dementia Rating
GDS-15: Geriatric Depression Scale
NPI: Neuropsychiatric Inventory
EQ-5D-5L: EuroQol five-dimension five-level questionnaire
