# Supplementary material for: Distribution of dementia severity and its functional correlates in community-dwelling older adults in Vietnam
Source: BMC Geriatr. 2026 May 2;26:862. doi: 10.1186/s12877-026-07588-3 (PMC13281577; doi:10.1186/s12877-026-07588-3)
Supplement: Supplementary file 1 — Supplementary Material 1. [file 12877_2026_7588_MOESM1_ESM.docx]

**Supplementary Material**

**Factors associated with dementia severity among community-dwelling older adults in Vietnam**

| **Factor** | **Very mild–moderate (CDR 0.5-2) (%)** | **Severe (CDR 3) (%)** | **p-value** |
| --- | --- | --- | --- |
| Age, mean ± SD (years) | 77.5 ± 10.0 | 85.4 ± 8.9 | <0.001 |
| Sex |  |  | 0.78 |
| Male | 82 (28.0) | 28 (26.4) |  |
| Female | 211 (72.0) | 78 (73.6) |  |
| Education |  |  | <0.001 |
| No formal education | 150 (51.2) | 82 (77.4) |  |
| Primary school | 56 (19.1) | 13 (12.3) |  |
| Secondary or higher | 87 (29.7) | 11 (10.4) |  |
| Occupation before retirement |  |  | 0.01 |
| Governmental staff | 49 (16.7) | 6 (5.7) |  |
| Farmer | 204 (69.6) | 89 (84.0) |  |
| Other | 40 (13.7) | 11 (10.4) |  |
| Marital status |  |  | <0.001 |
| Married, living with spouse | 205 (70.0) | 39 (36.8) |  |
| Widowed/single/divorced | 88 (30.0) | 67 (63.2) |  |
| BMI classification |  |  | 0.03 |
| Underweight | 60 (20.5) | 38 (35.8) |  |
| Normal | 214 (73.0) | 64 (60.4) |  |
| Overweight | 19 (6.5) | 4 (3.8) |  |
| Moderate-to-severe depression | 40 (13.7) | 41 (38.7) | <0.001 |
| Behavioral and psychological symptoms | 216 (73.7) | 98 (92.5) | <0.001 |
| High physical activity | 57 (19.5) | 5 (4.7) | <0.001 |
| IADL score, median (IQR) | 3 (0–7) | 0 (0–0) | <0.001 |
| EQ-5D-5L index, median (IQR) | 0.63 (0.48–0.84) | 0.03 (–0.51–0.42) | <0.001 |

**Supplementary Table S1. Distribution of participant characteristics by dementia severity**

Abbreviations: ADL = activities of daily living; IADL = instrumental activities of daily living; QoL = quality of life.

High physical activity defined as ≥600 MET-minutes/week according to the IPAQ-SF classification.

Depressive symptoms defined as GDS-15 score ≥6.

Behavioral and psychological symptoms assessed using the Neuropsychiatric Inventory (NPI).

IADL scores range from 0–8, with higher scores indicating greater independence.

EQ-5D-5L utility score ranges from 0–1, with higher scores indicating better quality of life.

| **Factor** | **Adjusted PR (95% CI)** | **p-value** |
| --- | --- | --- |
| **Age (years)** | 1.05–1.06 (range across models) | <0.001 |
| **Sex (Female vs Male)** | 0.91–1.12 (range across models) | >0.05 in all models |
| **Education** |  |  |
| Primary vs No education | 0.76 (0.41–1.40) | 0.383 |
| Secondary or above vs No education | 0.59 (0.30–1.17) | 0.132 |
| **Occupation** |  |  |
| Farmers vs Civil servants | 2.66 (1.13–6.24) | 0.025 |
| Others vs Civil servants | 1.98 (0.73–5.37) | 0.178 |
| **Marital status** |  |  |
| Single/Widowed/Divorced vs Married | 1.88 (1.22–2.90) | 0.004 |
| **BMI** |  |  |
| Normal vs Underweight | 0.86 (0.56–1.31) | 0.480 |
| Overweight (≥25) vs Underweight | 0.58 (0.21–1.63) | 0.304 |
| **Physical activity (IPAQ)** |  |  |
| Moderate vs Low | 0.86 (0.50–1.45) | 0.561 |
| High vs Low | 0.34 (0.12–0.93) | 0.036 |
| **Alcohol use (AUDIT)** |  |  |
| Mild risk to addicted vs Low risk | 1.39 (0.90–2.15) | 0.139 |
| **Nicotine dependence (FTND)** |  |  |
| Low–high vs Very low | 1.37 (0.92–2.05) | 0.120 |
| **Depression (GDS categories)** |  |  |
| Mild vs Normal | 1.15 (0.70–1.87) | 0.584 |
| Moderate/Severe vs Normal | 2.38 (1.52–3.73) | <0.001 |
| **Sleep disorder (PSQI categories)** |  |  |
| Minor vs No sleep disorder | 0.75 (0.41–1.37) | 0.349 |
| Moderate–Severe vs No sleep disorder | 1.65 (1.01–2.68) | 0.044 |
| **Neuropsychiatric symptoms (NPI, 1 vs 0)** |  |  |
| Present vs Absent | 2.86 (1.39–5.90) | 0.004 |
| **Social engagement score (continuous)** | 0.82 (0.73–0.91) | <0.001 |
| **Sleep quality score (PSQI, continuous)** | 1.54 (1.26–1.87) | <0.001 |
| **Depression score (GDS, continuous)** | 1.11 (1.05–1.17) | <0.001 |
| **Functional independence (IADL, continuous)** | 0.63 (0.53–0.75) | <0.001 |
| **Activities of daily living (ADL, continuous)** | 0.63 (0.55–0.72) | <0.001 |
| **Quality of life (EQ-5D index)** | 0.22 (0.15–0.34) | <0.001 |

**Supplementary Table S2. Minimally adjusted Poisson regression analyses examining associations with severe dementia (n = 399)**

| **Factors** | **VIF** | **1/VIF** |
| --- | --- | --- |
| Female (vs. male) | 1.47 | 0.68 |
| Age (per 1-year increase) | 1.07 | 0.94 |
| Widowed/single/divorced (vs. married) | 1.35 | 0.74 |
| Frequency of visiting friends/neighbors (per month) | 1.45 | 0.69 |
| Physical activity (MET-min/week) | 1.30 | 0.77 |
| Depressive symptoms (GDS-15 score) | 1.36 | 0.73 |
| Instrumental activities of daily living (per score increase) | 2.09 | 0.48 |
| Quality of life (EQ-5D-5L score increase) | 1.70 | 0.59 |
| Mean VIF | 1.47 |  |

Note: VIFs estimated from a linear probability model. Values below 5 indicate acceptable collinearity ([Kutner et al., 2004](https://www.researchgate.net/publication/344587293_Applied_Linear_Statistical_Models)).

**Supplementary Table S3. Variance inflation factors (VIF) for variables included in the multivariable Poisson regression model**

| **Factors** | **Mild - aRRR**  **(95% CI)** | **p-value** | **Moderate - aRRR (95% CI)** | **p-value** | **Severe - aRRR (95% CI)** | **p-value** |
| --- | --- | --- | --- | --- | --- | --- |
| Female (vs. male) | 1.02 (0.39–2.67) | 0.962 | 0.62 (0.24–1.64) | 0.338 | 0.67 (0.23–2.00) | 0.476 |
| Age (per 1-year increase) | 0.93 (0.88–0.98) | 0.004 | 0.97 (0.92–1.02) | 0.212 | 1.00 (0.94–1.06) | 0.985 |
| Widowed/single/divorced (vs. married) | 1.39 (0.45–4.32) | 0.572 | 4.08 (1.32–12.62) | 0.015 | 5.89 (1.70–20.36) | 0.005 |
| Frequency of visiting friends/neighbors (per month) | 1.00 (0.95–1.06) | 0.874 | 0.96 (0.90–1.02) | 0.164 | 0.89 (0.77–1.02) | 0.103 |
| Physical activity (MET-min/week) | 1.00 (1.00–1.00) | 0.750 | 1.00 (1.00–1.00) | 0.715 | 1.00 (1.00–1.00) | 0.512 |
| Depressive symptoms (GDS-15 score) | 1.18 (1.00–1.40) | 0.052 | 1.28 (1.08–1.51) | 0.005 | 1.36 (1.14–1.64) | 0.001 |
| Instrumental activities of daily living (per score increase) | 1.13 (0.95–1.35) | 0.177 | 0.84 (0.69–1.02) | 0.079 | 0.52 (0.38–0.73) | <0.001 |
| Quality of life (EQ-5D-5L score increase) | 0.66 (0.11–3.85) | 0.647 | 0.20 (0.04–1.11) | 0.066 | 0.04 (0.01–0.23) | <0.001 |

aRRR: adjusted relative risk ratio, adjusted for all covariates in the model. Reference category: very mild dementia (CDR = 0.5)

**Supplementary Table S4. Multinomial logistic regression analysis examining factors associated with different levels of dementia severity (reference: very mild dementia)**

| **Factors** | **Adjusted OR (95% CI)** | **p-value** |
| --- | --- | --- |
| Female (vs. male) | 0.84 (0.54–1.31) | 0.44 |
| Age (per 1-year increase) | 1.02 (0.99–1.04) | 0.18 |
| Widowed/single/divorced (vs. married) | 2.65 (1.66–4.23) | <0.001 |
| Frequency of visiting friends/neighbors (per month) | 0.98 (0.96–1.01) | 0.19 |
| Physical activity (MET-min/week) | 1.00 (0.99–1.00) | 0.46 |
| Depressive symptoms (GDS-15 score) | 1.09 (1.02–1.16) | 0.01 |
| Instrumental activities of daily living (per score increase) | 0.78(0.71–0.86) | <0.001 |
| Quality of life (EQ-5D-5L score increase) | 0.12 (0.06–0.24) | <0.001 |

Brant test for proportional odds assumption: p < 0.001 (assumption violated).

**Supplementary Table S5. Ordinal logistic regression analysis of factors associated with higher dementia severity**
